# HRD1 suppresses the growth and metastasis of breast cancer cells by promoting IGF-1R degradation

**DOI:** 10.18632/oncotarget.5733

**Published:** 2015-10-19

**Authors:** Yue-Mei Xu, Hong-Jiang Wang, Fang Chen, Wan-Hua Guo, Yan-Yang Wang, Hang-Yu Li, Jin-Hai Tang, Ying Ding, Ya-Chen Shen, Min Li, Wen-Ying Xuan, Lin-Hui Liu, Jia Wang, Xue-Rong Wang, Ze-Jun Gao, Xiu-Bin Liang, Dong-Ming Su

**Affiliations:** ^1^ State Key Laboratory of Reproductive Medicine, Department of Pathology, Nanjing Medical University, Nanjing, China; ^2^ Department of Breast Surgery, First Affiliated Hospital of Dalian Medical University, Dalian, China; ^3^ Key Laboratory of Human Functional Genomics of Jiangsu Province, Nanjing Medical University, Nanjing, China; ^4^ Department of Nuclear Medicine, The Affiliated Drum Tower Hospital of Nanjing University, Nanjing, China; ^5^ Department of General Surgery, 4th Affiliated Hospital, China Medical University, Shenyang, China; ^6^ Department of General Surgery, The Affiliated Jiangsu Cancer Hospital, Nanjing Medical University, Nanjing, China; ^7^ Center of Metabolic Research, Nanjing Medical University, Nanjing, China; ^8^ Center of Cellular therapy, the Second Affiliated Hospital of Nanjing Medical University, Nanjing, China

**Keywords:** HRD1, breast cancer, metastasis, IGF-1R, degradation

## Abstract

HRD1 (3-hydroxy-3-methylglutaryl reductase degradation) is an E3 ubiquitin ligase. We found that HRD1 was significantly downregulated in 170 breast cancer tissues. Low tumoral HRD1 expression was correlated with clinicopathological characteristics and a shorter survival in breast cancer patients. P65 specifically bound to the HRD1 promoter and inhibited HRD1 expression. Suppression of NF-κB activity reversed IL-6-induced downregulation of HRD1 expression. HRD1 interacted with IGF-1R and promoted its ubiquitination and degradation by the proteasome. Overexpression of HRD1 resulted in the inhibition of growth, migration and invasion of breast cancer cells *in vitro* and *in vivo*. Furthermore, HRD1 attenuated IL-6-induced epithelial-mesenchymal transition in MCF10A cells. These findings uncover a novel role for HRD1 in breast cancer.

## INTRODUCTION

Breast cancer is the most common malignant disease and the leading cause of cancer-related deaths among women all over the world [1]. Advances in surgery, radiotherapy, hormonal therapy and chemotherapy have improved the treatment of breast cancer, and yet more than 410,000 women still die from this disease every year [2]. The high breast cancer-related mortality rates are associated with tumor invasion and metastasis. Indeed, invasive breast cancer is the main pathologic type worldwide and over 90% of the deaths of breast cancer patients are due to metastases [3]. However, our understanding of the molecular mechanisms underlying breast cancer invasion and metastases remains incomplete.

The epithelial-mesenchymal transition (EMT) is a biological process characterized by a loss of polarity in epithelial cells and their assumption of a mesenchymal cell phenotype. The EMT has been implicated in tumor growth and metastasis. As tumors progress, an oncogenic EMT can result in increased migratory and invasive capabilities of tumor cells, which may in turn contribute to metastatic dissemination and tumor progression [4]. The cellular and molecular changes occurring during EMT-mediated tumor progression of various carcinomas include downregulation of E-cadherin expression and upregulation of expression of non-epithelial cadherins, such as N-cadherin, Vimentin, Snail and Twist [5].

In breast cancer, the induction and maintenance of EMT and tumor progression is greatly affected by the inflammation microenvironment [6, 7]. For example, the inflammatory cytokines such as TNF-α, IL-1β and IL-6 induce EMT properties by activating the NF-κB pathways [8]. Emerging evidence indicates a correlation between that EMT marker expression and poor prognosis, while histopathologic changes in the EMT are associated with a high tumor grade, high mitotic index, and negative estrogen/progesterone-receptor status [9–12]. Therefore, interest is growing in determining how and what is changed in breast cancer cells during the inflammation-induced EMT.

An involvement of the insulin-like growth factor (IGF) family is implicated in tumorigenesis of breast cancer. The type 1 IGF receptor (IGF-1R) is often overexpressed in breast cancer, and this overexpression has been associated with worse prognosis and shorter disease-free survival [13, 14]. Overexpression and hyperphosphorylation of IGF-1R is also associated with resistance to several anticancer treatments, including endocrine therapy, anti-human epidermal growth factor receptor 2 (Her2) therapy and chemotherapy [15]. Breast cancer cells are thought to have a defect in IGF-1R ubiquitination, which is a necessary step for the degradation of this receptor, but the underlying mechanism is not clearly understood [16, 17]. Nevertheless, inhibiting of IGF-1R expression and enhancing its degradation is considered as a promising potential therapeutic strategy for the treatment of breast cancer [18].

HRD1 (3-hydroxy-3-methylglutaryl reductase degradation), which is also called synoviolin, is an ER-associated degradation (ERAD)-associated E3 ubiquitin ligase that functions by promoting degradation of misfolded proteins in processes such as embryogenesis and rheumatoid arthritis [19, 20]. Although, HRD1 has been shown to increase the ubiquitination and degradation of p53, little is known about the role of HRD1 itself in tumors [21]. In this study, we examined the expression of HRD1 in breast cancer and investigated its function in degradation of IGF-1R, in order to better understand its role in tumorigenesis of breast cancer and its potential implications for cancer therapy.

## RESULTS

### HRD1 is downregulated in breast cancer

**Figure 1:**
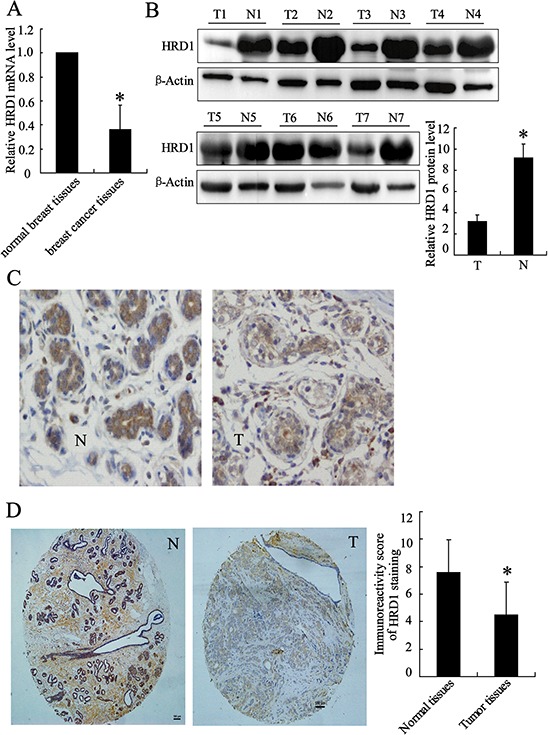
HRD1 was downregulated in breast cancer versus non-cancer tissues **A.** HRD1 mRNA level was determined in breast cancer tissue specimens (*n* = 7) and matched adjacent normal breast tissues (*n* = 7) by real time PCR. **B.** HRD1 protein levels in patients as in (A) were measured by Western blotting. **C.** Representative images of immunohistochemical staining of tissues with HRD1 antibody. N: normal, T: tumor; original magnification, ×200. **D.** Immunohistochemical staining of tissue microarrays with HRD1 antibody; original magnification, ×100. Immunoreactivity score of HRD1 staining was available from 170 pairs of tissues. *P < 0.05, compared to the normal breast tissues.

The expression levels of HRD1 were investigated in breast cancer tissue specimens (*n* = 7) and matched adjacent normal breast tissues (*n* = 7) using realtime PCR and western blotting (Figure 1A, 1B). Immunohistochemical staining of the breast tissues indicated a predominant localization of HRD1 in the cytoplasm of the breast cancer and normal cells. The expression of HRD1 was significantly decreased in breast cancer cells (Figure 1C). These results were confirmed by TMA of breast cancer patients, which showed a significant reduction of HRD1 in breast cancer tissues when compared with matched normal breast tissues (Figure 1D, *p* < 0.01).

### Downregulation of HRD1 expression is correlated with clinicopathological characteristics and a shorter survival in breast cancer patients

**Figure 2:**
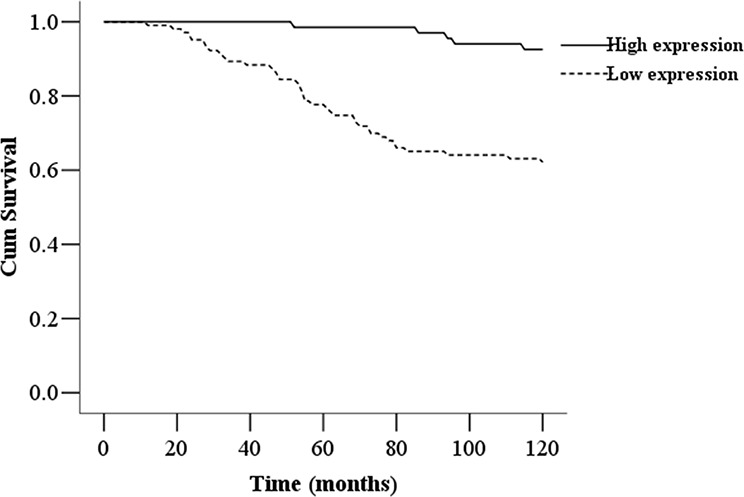
Kaplan-Meier survival curves of breast cancer patients The 10-year overall survival rate of breast cancer patients (*n* = 170) with low HRD1 expression was significantly lower than that of breast cancer patients with high HRD1 expression (*P* < 0.01).

We investigated the expression levels of HRD1 in 170 patients with breast cancer and examined their associations with clinicopathological factors and overall survival. The expression levels of HRD1 in breast cancer patients were significantly correlated with IGF-1R status, breast cancer grade and lymph node metastasis (*p* < 0.05). However, HRD1 expression in breast cancer tissues was not associated with patient ages, tumor size, tumor histology and subtypes, ER status, PR status, or HER2 status (Table 1). Moreover, Life Table analysis revealed that low HRD1 staining was significantly correlated with a poorer overall 10 year survival of all breast cancer patients (*p* < 0.001, log rank test; Figure 2).

**Table 1:** Correlation of clinicopathological features of breast cancer with HRD1 expression levels

Characteristics	All cases	HRD1 expression levels		
		High expression	Low expression	*P* value
Age				0.552
<50	86	32	54	
≥50	84	35	49	
Tumor size (cm)				0.225
<2	74	33	41	
≥2	96	34	62	
Grade				0.023*
I, well-differentiated	23	14	9	
II, moderately differentiated	99	40	59	
III, poorly differentiated	48	13	35	
Tumor histological				0.064
Ductal carcinoma *in situ*	23	14	9	
Invasive ductal carcinoma	130	48	82	
Invasive lobular carcinoma	17	5	12	
ER status				0.989
Negative	61	24	37	
Positive	109	43	66	
PR status				0.557
Negative	79	33	46	
Positive	91	34	57	
HER2/neu status				0.776
Negative	112	45	67	
Positive	58	22	36	
IGF-1R status				0.000*
Negative	57	36	21	
Positive	113	31	82	
Lymph node-metastasis				0.000*
Negative	90	57	33	
Positive	80	10	70	
Nuclear p65 expression				
High expression	60	37	23	0.000*
Low expression	110	30	80	

### The expression of HRD1 was downregulated by NF-κB activation

**Figure 3:**
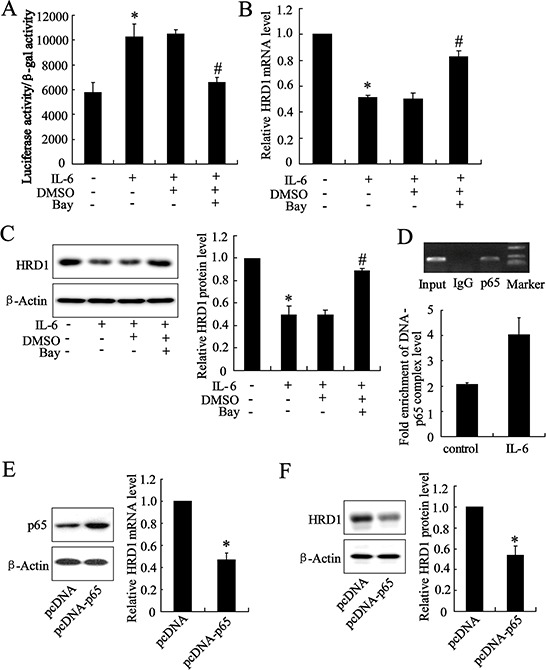
The expression of HRD1 was downregulated by NF-κB activation MCF-7 cells were pretreated with Bay 11–7082 (5 μmol/L) for 2 h, and treated with IL-6 (50 ng/ml) for another 24 h. Then, the NF-κB transcriptional activity **A.** HRD1 mRNA level **B.** and protein level **C.** were then measured. **D.** P65 bound to the HRD1 promoter in MCF-7 cells in a ChIP analysis. ChIP-qPCR analysis was performed to measure the capacity of p65 binding to HRD1 promoter. The plasmid of pcDNA-P65 was transfected into MCF-7 cells for 48 h, and then, the HRD1 mRNA level **E.** and protein level **F.** were measured. *P < 0.05, compared to control. #*P* < 0.05, compared to IL-6 treatment.

The Genomatix databases predicted that NF-κB could bind to the HRD1 gene promoter. We explored the possible involvement of NF-κB in inhibition of HRD1 expression in breast cancer cells by treating MCF-7 cells with IL-6. The IL-6 treatment significantly increased NF-κB activity (Figure 3A) but decreased HRD1 expression at the mRNA level (Figure 3B). This IL-6 induced downregulation of HRD1 expression was abolished by Bay 11–7082 (Figure 3C and Supplementary Figure S3A). Furthermore, the specifically association of P65, the subunit of NF-κB, and HRD1 promoter was confirmed by Chromatin immunoprecipitation (ChIP) assays (Figure 3D). In addition, IL-6 treatment increased p65 binding with HRD1 promoter. Overexpression of p65 clearly reduced HRD1 expression (Figure 3E, 3F). These results indicated that NF-κB activation is responsible for the downregulation of HRD1 expression in breast cancer cells.

### HRD1 promotes IGF-1R ubiquitination for degradation

**Figure 4:**
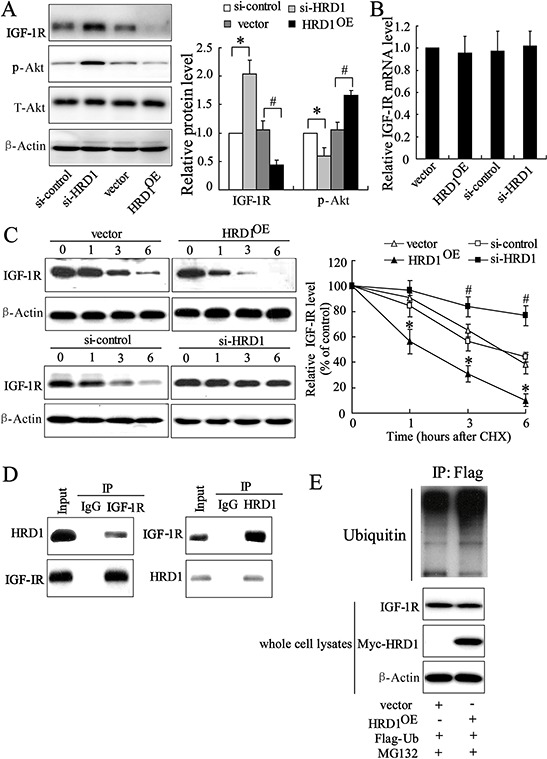
HRD1 promotes IGF-1R ubiquitination for degradation **A.** The protein levels of HRD1, IGF-1R and the downstream target *P*-Akt were measured by immunoblotting in HRD1 overexpressing (HRD1^OE^), HRD1 siRNA (si-HRD1) and their respective control transfected MCF-7 cells. **B.** Total mRNA was prepared from HRD1^OE^, si-HRD1 and their respective control transfected MCF-7 cells and IGF-1R mRNA levels were quantified using real time RT-PCR. **C.** MCF-7 cells were transfected with HRD1^OE^, si-HRD1 and their respective control for 48 h, followed by exposure to cycloheximide (CHX 50 mg/ml) for 0, 1, 3, or 6 h. the protein of IGF-1R and HRD1 in whole cell lysates was measured by immunoblotting. The intensity of the IGF-1R protein bands was analyzed by densitometry, after normalization to the corresponding β-Actin level. **D.** MCF-7 cells were pretreated with MG132 (10 μM) for 6 h and endogenous protein-protein interactions between HRD1 and IGF-1R were determined by immunoprecipitation (IP) with HRD1 or IGF-1R antibodies, followed by immunoblotting. IgG was used as a negative control for IP. **E.** Ubiquitination of IGF-1R was induced by HRD1. Flag-ubiquitin was coexpressed in MCF-7 cells with myc-HRD1 or vector control with treatment of MG132 (10 μM) for 6 h. Ubiquitinated IGF-1R protein was immunoprecipitated using Flag-Tag antibody and further detected with Anti-IGF-1R antibody. The endogenous IGF-1R and myc-HRD1 in the whole cell lysates were examined by anti-IGF-1R and anti-myc antibodies. *P < 0.05, compared to vector. #*P* < 0.05, compared to si-control.

Xu et al reported that IGF-1R expression level was significantly increased in breast cancer tissues [22]. We also observed that IGF-1R expression level was negatively correlated with the expression levels of HRD1 (correlation = − 0.507, *P* < 0.01) in the breast cancer tissues, indicating a potential relationship between IGF-1R and HRD1. Overexpression of HRD1 inhibited IGF-1R expression at the protein level and AKT phosphorylation, whereas HRD1-specific siRNA increased IGF-1R expression levels and AKT phosphorylation in MCF-7 cells (Figure 4A and Supplementary Figure S1A). Besides, HRD1 overexpression significantly attenuated Akt activation induced by IGF (Supplementary Figure S1C). In contrast, upregulation or downregulation of HRD1 expression had no effect on IGF-IR mRNA levels (Figure 4B).

Next, we further explored the potential mechanisms relevant to the decrease of HRD1 on IGF-1R. Treatment of MCF-7 cells with cycloheximide (CHX), an inhibitor of protein synthesis, resulted in promotion of IGF-1R degradation under the situation of HRD1 overexpression. In contrary, this degradation was suppressed by HRD1 knockdown (Figure 4C). A physical interaction between HRD1 and IGF-1R was evident in the co-immunoprecipitation (co-IP) analysis in endogenous settings (Figure 4D). We also found an elevation in ubiquitinated IGF-1R expression when inhibited its degradation by MG132, while HRD1 overexpression further increased the ubiquitination of IGF-1R (Figure 4E). These results indicated that HRD1 served as an E3 ligase that promoted IGF-1R ubiquitination for degradation by the proteasome.

### HRD1 inhibits *in vitro* growth, migration, and invasion of breast cancer cells

**Figure 5:**
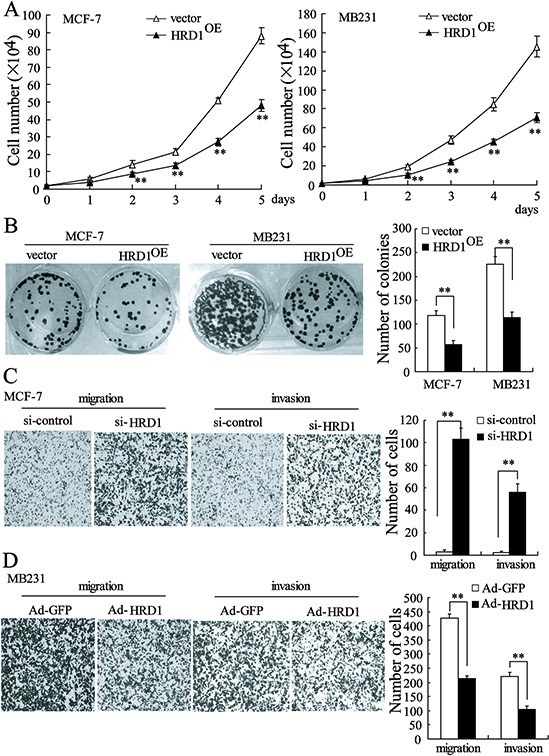
HRD1 inhibits growth, colony formation, migration, and invasion of breast cancer cells MCF-7 and MB231 cells stably expressing HRD1 were used to perform cell MTT assay **A.** and colony-formation assay **B. C.** MCF-7 cells were transfected with si-control or si-HRD1 for 48 h prior to the transwell assays. **D.** MB231 cells were infected with Ad-GFP or Ad-HRD1 for 48 h, and then transwell assays were performed. **P < 0.01, compared to vector or si-control.

We assessed the biological role of HRD1 in breast cancer by investigating the effects of HRD1 over-expression on the viability and colony formation of MCF-7 and MB231 cells. The results obtained from MTT assay confirmed that the growth of MCF-7 and MB231 cells was clearly inhibited by HRD1 overexpression (Figure 5A). The cells stably over-expressing HRD1 formed fewer colonies when compared with vector control cells (Figure 5B). We next measured MCF-7 and MB231 cell invasion and migration with Transwells. Downregulation of HRD1 expression significantly increased migration and invasion of MCF-7 cells when compared with controls (Figure 5C and Supplementary Figure S1B). The MB231 cells with increased expression of HRD1 showed significantly inhibited migration and invasion (Figure 5D). However, we also found that overexpression of a dominant-negative HRD1 mutant (C291S) had no effect on MB231 cell growth and migration, and invasion (Supplementary Figure S2A and S2B). These results indicated that HRD1-mediated IGF-1R degradation was dependent of HRD1 enzyme activity.

### Overexpression of HRD1 in breast cancer cell inhibits *in vivo* tumor growth and metastasis

**Figure 6:**
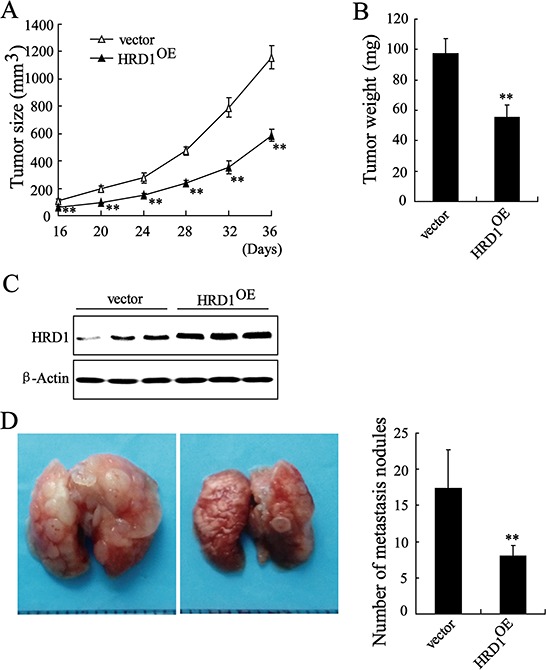
Overexpression of HRD1 in breast cancer cells inhibits *in vivo* tumor growth and metastasis MB231 cells stably overexpressing of HRD1 and the corresponding controls were injected into the right flank and left flank of nude mice, respectively. Tumor sizes were monitored twice per week **A.** and tumor weights **B.** were measured. **C.** The expression of HRD1 in tumor lysates was measured. **D.** MB231 cells stably overexpressing of HRD1 were inoculated into nude mice of via the tail vein. After 5 weeks, the lungs from mice in each experimental group were examined for calculation of the numbers of tumor nodules on lung surfaces. **P < 0.01, compared to vector.

We validated the *in vivo* effects of HRD1 on the growth of breast cancer cells by injecting MB231 cells stable overexpressing HRD1 or the corresponding controls into the right flank and left flank of nude mice, respectively. Sixteen days after injection, we found that the tumors formed in the HRD1-overexpressing group were significantly smaller than those in the vector group (Figure 6A). As shown in Figure 6B, the mean tumor weight was dramatically lower in the HRD1-overexpressing group (97.5 ± 9.5 mg) compared to the vector group (55.5 ± 7.7 mg). The overexpression of HRD1 in tumor lysates was also confirmed (Figure 6C). We then inoculated MB231 cells stably overexpressing HRD1 into nude mice via the tail vein to explore the *in vivo* effects of HRD1 on the metastasis of breast cancer cells. Increased HRD1 expression led to a decrease in the number of metastatic nodules when compared with the control group (Figure 6D).

### HRD1 inhibited breast cancer cell EMT

**Figure 7:**
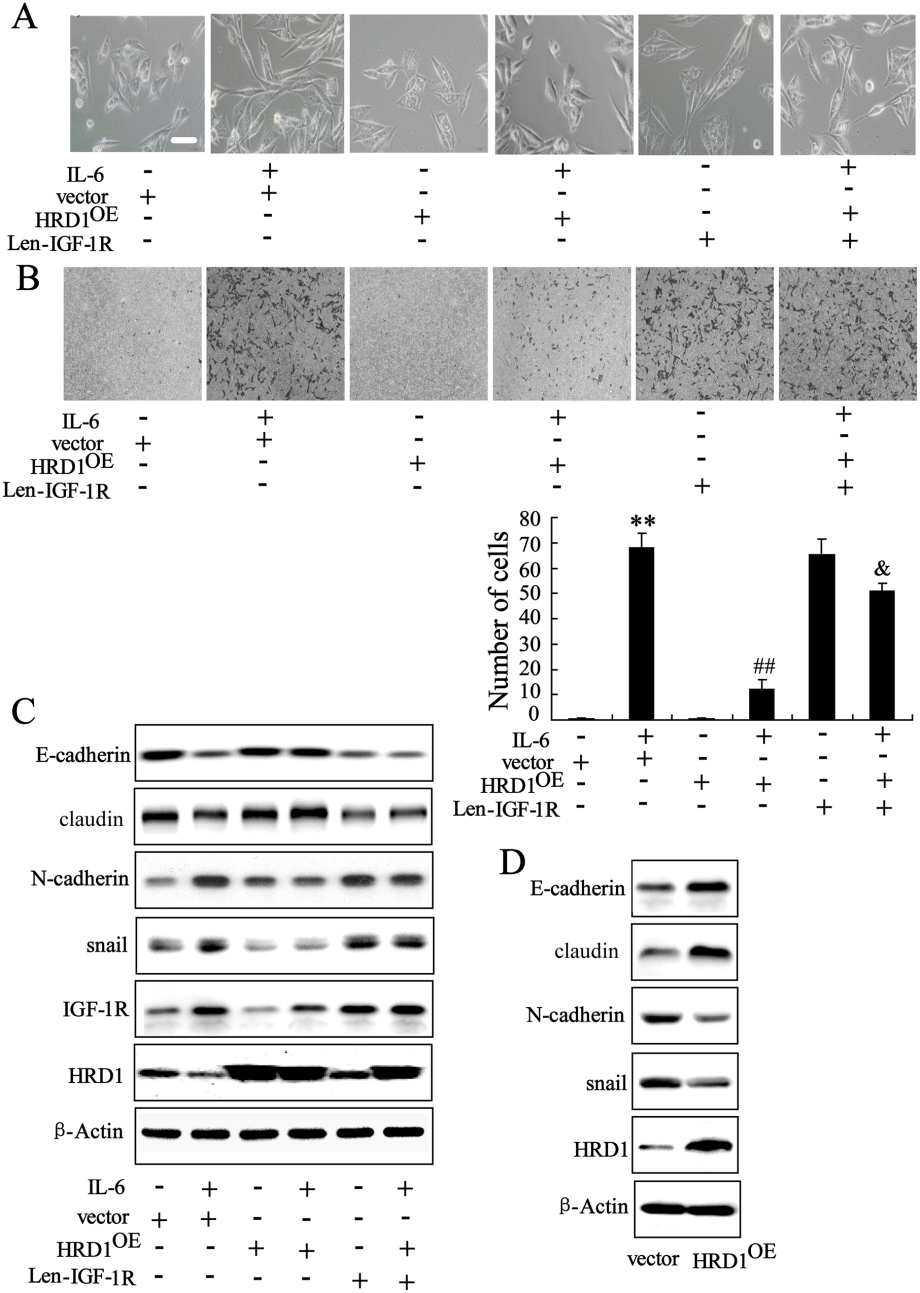
HRD1 is implicated in breast cancer cell EMT MCF10A cells stably expressing HRD1 were infected with lentivirus of IGF-1R for 48 h, and then treated with or without IL-6, followed by phase contrast microscopy **A.** transwell **B.** and western blotting **C. D.** MCF-7 cells stably expressing HRD1 were examined by western blotting. Scale bar represents 100 μm. **P < 0.01, compared to control. ##*P* < 0.01, compared to IL-6+vector. &*P* < 0.01, compared to IL-6+HRD1^OE^.

Increasing evidence supports an involvement of the EMT in tumor growth and metastasis [12]. In this study, we investigated whether HRD1 expression is implicated in the EMT. We found that IL-6 treatment induced a morphological change in MCF10A cells to the fibroblast-like scattered morphology of mesenchymal cells; this change be reversed by overexpression of HRD1. Interestingly, upregulation of IGF-IR abolished the effect of HRD1 overexpression on EMT in MCF10A cells treated with IL-6 (Figure 7A). Besides, HRD1 overexpression decreased the migration of MCF10A cells treated with IL-6, which were reversed by upregulation of IGF-1R expression (Figure 7B). Since HRD1 was not found having significant impact on the proliferation of cancerous cell when cultured in the serum-free medium for 24 hours (Supplementary Figure S4), we considered this inhibition of cell migration a direct effect from HRD1 rather from the secondary decreased cell number resulting from HRD1 overexpression. Western blotting also revealed that HRD1 overexpression reversed the decrease expression of epithelial markers such as E-cadherin, Claudin-1 and the increase in the mesenchymal markers such as N-cadherin and snail induced by IL-6, but this phenotype was reversed by IGF-1R (Figure 7C). Overexpression of HRD1 in MCF-7 cells resulted in increased expression of E-cadherin and Claudin-1, and decreased expression of N-cadherin as well as snail (Figure 7D).

## DISCUSSION

This study identified the E3 ubiquitin ligase, HRD1, as an inhibitor of the growth and metastasis of breast cancer. A current model of the triggering of the EMT by decease of HRD1 and increase of IGF-1R is shown in Figure 7. HRD1 expression levels are downregulated in breast cancer cells due to the activation of NF-κB. IGF-1R known as an oncoprotein is directly suppressed by HRD1, and downregulation of HRD1 increases IGF-1R expression levels in breast cancer.

We demonstrated, for the first time, that HRD1 is a transcritional target of p65, which is a key regulator of breast cancer cell growth and metastasis [23, 24]. Our data showed that NF-κB could directly bind to the HRD1 promoter and overexpression of the subunit of NF-κB p65 markedly reduced HRD1 expression. Notably, IL-6 treatment led to an increase in NF-κB activity and a decrease in HRD1 expression, while Bay 11–7082, an inhibitor of NF-κB, reversed the inhibition of HRD1 expression induced by IL-6. Additionally, TNF-ɑ also was found inhibited HRD1 expression, which could be abolished by Bay 11-7082 as well (Supplementary Figure S3B). Based on these finding, we hypothesized that NF-κB activation played a direct role on downregulation of HRD1 expression in breast cancer.

In our current study, a significantly lower HRD1 expression was observed in breast cancer than in non-cancer tissues. Specifically, HRD1 expression was significantly decreased in tumors with poorly differentiated grade and undergoing extensive metastasis. A shorter survival was also observed in breast cancer patients with lower HRD1 expression than those with higher HRD1 expression levels. We also demonstrated that overexpression of HRD1 led to a significant decrease *in vitro* cell viability, colony formation, migration, and invasion, as well as tumor growth and metastasis *in vivo*. Downregulation of HRD1 expression promoted MCF-7 cell migration and invasion. These findings implicate that HRD1 was involved in the progression and metastasis of breast cancer, indicating that HRD1 may excert a novel prognostic or progression marker for breast cancer in the future.

Activation of IGF-1R plays a crucial role in breast cancer progression and metastasis. The significantly increased IGF-1R expression level was reported in breast cancer tissues [13, 25]. Targeting IGF-IR expression and degradation is therefore a potential therapeutic strategy for the treatment of breast cancer [18]. In the current study, we demonstrated that HRD1 downregulated IGF-1R expression and therefore downstream AKT activity [22]. Furthermore, we found that HRD1 could interact with IGF-IR acting as a ubiquitin E3 ligase that targeted IGF-1R for degradation via the ubiquitin proteasome pathway. This novel observation obtained from *in vitro* study was further evidenced clinically by an inversed correlation between IGF-1R and HRD1 expression in breast cancer tissues. These data, for the first time, revealed the regulation of HRD1 on IGF-1R expression is through proteasomal degradation pathway.

Activation of the IL6/NF-κB signaling pathway in non-transformed MCF10A cells induces the EMT, a driver of tumor growth and metastasis [26, 27]. We demonstrated that HRD1 overexpression abolished the IL-6 induced EMT in MCF10A cells. In contrast, upregulation of IGF-1R resulted in a surprising reversal of the effects of overexpression of HRD1 on the EMT (Figure 7), indicating the impact of HRD1 on EMT was through IGF-1R signaling pathway. The EMT, as a key differentiation process, is involved in tumor cell growth and metastasis [28]. Our data further demonstrate that overexpression of HRD1 in MCF-7 cells resulted in MET. Therefore, ubiquitination and degradation of IGF-1R mediated by HRD1 represents an inhibitory mechanism for IL-6-induced signaling that is critical for induction of EMT.

In conclusion, our findings indicated that HRD1 expression was negatively correlated with breast cancer progression and that loss of HRD1 was therefore a prognostic marker of poor survival in breast cancer patients. NF-κB activation was responsible for the downregulation of HRD1 in breast cancer cells. Overexpression of HRD1 prevented the formation of breast cancer cell malignant phenotypes and, importantly, suppressed the EMT by degradation of IGF-1R. Based on our findings, we proposed that restoration of HRD1 expression may be a novel strategy for human breast cancer therapy.

## MATERIALS AND METHODS

### Cell lines and antibodies

Human breast cancer (MCF-7, MDA-MB-231), mammary epithelial (MCF10A), and embryonic kidney 293T (HEK293T) cell lines were obtained from ATCC. The cells were cultured at 37°C in a humidified atmosphere containing 95% air and 5% CO_2_. Antibodies against IGF-1R, AKT, *p*-AKT, E-cadherin, claudin, N-cadherin, snail and p65 were purchased from Cell Signaling Technology (Beverly, MA, USA). HRD1 antibody for Western blot analysis was obtained from Sigma Aldrich (St. Louis, MO, USA). HRD1 antibody for IHC was purchased from Abgent (San Diego, Califonia, USA). Antibodies against β-Actin, myc-Tag and HA-Tag were acquired from Santa Cruz Biotechnology (Santa Cruz, CA, USA).

### Patients and tissue microarray construction

The breast cancer samples were obtained from 170 patients undergoing surgical resection at the Second Affiliated Hospital of Nanjing Medical University, Nanjing, China, between 2001 and 2004. Six patients lacked five years of follow-up time and were therefore excluded from the 5-year survival analysis, but not from the multivariate analysis. The clinico-pathological characteristics of patients with breast cancer are compiled in Table 1. Tissue microarrays (TMAs) containing 0.6 mm^2^ cores from each tumor, identified by two pathologists, were constructed as described in a previous report [29]. The utilization of the tumor material for research purposes was approved by the ethical committees at Nanjing Medical University.

### Immunohistochemistry (IHC)

TMA blocks (18 tumors per block, 4 cores of 1.0 mm per tumor) were constructed using an automated TMA station (Galileo TMA CK3500, ISE, Milan, Italy). All sections were cut shortly prior to immunostaining. The expression of HRD1 was measured using IHC as previously described [30]. Staining of HRD1 in tissue was scored independently by two pathologists blinded to the clinical data, by applying a semiquantitative immunoreactivity score (IRS) in the training cohort, as described previously [31]. Under these conditions, samples with IRS 0–5 and IRS 6–12 were classified as low and high expression of HRD1, respectively. Additionally, samples with IRS ≤4 and IRS >4 were classified as low and high expression of nuclear p65, respectively. After establishing the immunohistochemical assessment criteria in the training cohort, expression of HRD1 in the validation cohort was scored using the same procedure.

### Stable cell lines

MCF10A, MCF-7 and MDA-MB-231 cells were stably transduced with EGFP or EGFP-tagged HRD1 lentivirus according to the manufacturer's instructions (GENECHEM, Shanghai, China). Transfection of MCF10A, MCF-7 and MDA-MB-231 cells was done at a 10-fold multiplicity of infection virus particle concentration. The expression level of HRD1 was measured by western blotting.

### Small interfering RNA

Small interfering RNA specific for HRD1 (si-HRD1) and control siRNA (si-control) was synthesized (Ribobio, Guangzhou, China) and transfected using Lipofectamine 2000. The sequences of siRNA were: 5′-CCAUGAGGCAGUUCAAGAAdTdT-3′ and 3′-dTdTGGUACUCCGUCAAGUUCUU-5′.

### Real-time PCR assay

The mRNA was quantified by real-time PCR using a LightCycler480 II Sequence Detection System (Roche, Basel, Switzerland). Primers used to identify HRD1 were: forward, 5′-AACCCCTGGGACAACAAGG-3′ and reverse, 5′-GCGAGACATGATG GCATCTG-3′. β-Actin was used as a internal control: forward, 5′-GCAAGTGCTTC TAGGCGGAC-3′ and reverse, 5′-AAGAAAGGGTGTAAAACGCAGC-3′.

### Transient transfection and luciferase reporter assay

Transcriptional activity of NF-κB was assessed in MCF-7 cells using the NF-κB luciferase reporter and a plasmid containing the β-galactosidase gene driven by the cytomegalovirus promoter. Twenty-four hours after transfection, MCF-7 cells were treated with IL-6 for an additional 24 h. The cells were then incubated and harvested for luciferase reporter assays. Luciferase activity was determined as our previous report [32].

### Western blot analysis

Cells were washed twice in ice-cold PBS, and then solubilized in RIPA lysis buffer (Vazyme, Nanjing, China). Samples containing equal amounts of protein were analyzed by western blotting as previously reported [32].

### Animal tumor model

Female athymic nude mice (6-weeks-old) were purchased from Shanghai Laboratory Animal Centre (Chinese Academy of Sciences, Shanghai, China) and maintained in cage housing under specific pathogen-free conditions. Cultured MDA-MB-231 cells transfected with EGFP or EGFP-tagged HRD1 lentivirus were harvested from 6-well plates and resuspended in 0.2 ml of PBS at 5 × 10^7^ cells/ml. Cells were injected either into the right or left flank region of the mice to generate the orthotopic model or into tail vein of the mice to generate the lung metastasis model. The mice (ten mice per group) were sacrificed 6 weeks after the injection. The size and weight of the lungs were assessed, and visible tumors on the lung surface were counted. This study was carried out in accordance with the guidelines of the Institutional Animal Care and Use Committee at Nanjing Medical University and was approved by the Committee on the Ethics of Animal Experiments of Nanjing Medical University.

### Co-immunoprecipitation (Co-IP)

The MCF-7 cells were grown to confluence and processed for Co-IP by standard procedures, as previously reported [33]. Briefly, the cells were harvested and lysed in cold lysis buffer [50 mM Tris [pH 7.4], 150 mM NaCl, 1 mM EDTA, 0.5% (v/v) NP-40, 10% (v/v) glycerol, 1 mM PMSF, and a complete protease inhibitor cocktail tablet]. The cell extracts were incubated with protein A/G agarose beads or control IgG (Santa Cruz,) as a pretreatment. The lysates were then incubated with anti-HRD1 antibody, anti-IGF-1R antibody, anti-HA antibody or control IgG for 1 h, followed by incubation overnight with protein A/G agarose beads. The beads were collected by centrifugation, washed three times with the lysis buffer and resuspended in 1 × SDS loading buffer. The immunoprecipitates were eluted from the beads by incubation at 95°C for 5 min. The eluted proteins were separated by SDS-PAGE and western blotting was subsequently performed with indicated antibodies.

### Migration, invasion, cell viability and colony-formation assays

Stable cell lines of MCF-7 and MB231 cells were seeded in 96-well dishes at 1 × 10^4^ cells per well for different times. Cell viability was determined using MTT assays. The MCF-7 and MB231 cells were seeded in 6-well dishes at 200 cells per well and infected with HRD1 lentivirus for 3 weeks. Colonies with a diameter of more than 100 μm were counted.

For the migration assays, after 48 h transfection, 1 × 10^5^ cells in serum-free media were placed into the upper chamber of a Transwell insert (8-μm pore size; Millipore). For the invasion assays, cells in serum-free medium were placed into the upper chamber of an insert coated with Matrigel (Sigma-Aldrich). Medium containing 10% FBS was added to the lower chamber. After incubation for 24 h, the cells remaining on the upper membrane were removed with cotton wool. Cells that had migrated or invaded through the membrane were fixed in methanol, stained with crystal violet (0.04% in water; 100 μl), counted using an inverted microscope and photographed.

### Chromatin immunoprecipitation (ChIP) assay

ChIP assay was performed using the ChIP assay kit (Upstate Biotechnology) following the manufacturer's protocol. Briefly, MCF-7 cells were harvested and fixed in 1% (v/v) formaldehyde for 10 min at room temperature. Then, cells were lysed in SDS lysis buffer and the chromatin was sonicated. The chromatin was incubated overnight at 4°C with anti-p65 antibody, and normal IgG as a negative control. The sequences of PCR using primers framing the HRD1 promoter region of interest were synthesized: forward, 5′-TGGAAGCAGAGGGACCCAGGACC-3, reverse, 5′-AGTGAAAGCAGGAGAAGGGACGG-3′. To examine whether IL-6 could increase the binding activity of p65 on the promoter of HRD1, we used chromatin immunoprecipitation and quantitative real-time PCR (ChIP-qPCR) assay. For every promoter studied, a ΔCt value was calculated for each sample: ΔCt = Ct (sample)-Ct (Input). Next, a ΔΔCt value was calculated: ΔΔCt = ΔCt (sample immunoprecipitated with p65 antibody) - ΔCt (sample immunoprecipitated with IgG). The fold difference between p65 antibody-immunoprecipitated samples and those immunoprecipitated with IgG was calculated using 2^−ΔΔCt^.

### Statistical analysis

Statistical analyses were performed using statistical analysis software SPSS 13.0. Data were expressed as the mean ± SD. Analysis of variance (ANOVA) was used to determine the statistical differences among the groups. The Kaplan-Meier method was used to estimate survival rates. A multivariate analysis of the independent prognostic factors was conducted using the Cox proportional hazards model. A *P* value of less than 0.05 and are provided in the figures. A *P* value <0.05 was considered statistically significant.

## SUPPLEMENTARY FIGURES



## References

[1] Baselga J, Norton L (2002). Focus on breast cancer. Cancer Cell.

[2] Coughlin SS, Ekwueme DU (2009). Breast cancer as a global health concern. Cancer Epidemiol.

[3] Padmore RF, Fowble B, Hoffman J, Rosser C, Hanlon A, Patchefsky AS (2000). Microinvasive breast carcinoma: clinicopathologic analysis of a single institution experience. Cancer.

[4] Yilmaz M, Christofori G (2009). EMT, the cytoskeleton, and cancer cell invasion. Cancer Metastasis Rev.

[5] Zhou XM, Zhang H, Han X (2014). Role of epithelial to mesenchymal transition proteins in gynecological cancers: pathological and therapeutic perspectives. Tumour Biol.

[6] Leibovich-Rivkin T, Liubomirski Y, Bernstein B, Meshel T, Ben-Baruch A (2013). Inflammatory factors of the tumor microenvironment induce plasticity in nontransformed breast epithelial cells: EMT, invasion, and collapse of normally organized breast textures. Neoplasia.

[7] Sullivan NJ, Sasser AK, Axel AE, Vesuna F, Raman V, Ramirez N, Oberyszyn TM, Hall BM (2009). Interleukin-6 induces an epithelial-mesenchymal transition phenotype in human breast cancer cells. Oncogene.

[8] Soria G, Ofri-Shahak M, Haas I, Yaal-Hahoshen N, Leider-Trejo L, Leibovich-Rivkin T, Weitzenfeld P, Meshel T, Shabtai E, Gutman M, Ben-Baruch A (2011). Inflammatory mediators in breast cancer: coordinated expression of TNFα & IL-1β with CCL2 & CCL5 and effects on epithelial-to-mesenchymal transition. BMC Cancer.

[9] Tomaskovic-Crook E, Thompson EW, Thiery JP (2009). Epithelial to mesenchymal transition and breast cancer. Breast Cancer Res.

[10] Foroni C, Broggini M, Generali D, Damia G (2012). Epithelial-mesenchymal transition and breast cancer: role, molecular mechanisms and clinical impact. Cancer Treat Rev.

[11] Drasin DJ, Robin TP, Ford HL (2011). Breast cancer epithelial-to-mesenchymal transition: examining the functional consequences of plasticity. Breast Cancer Res.

[12] Gunasinghe NP, Wells A, Thompson EW, Hugo HJ (2012). Mesenchymal-epithelial transition (MET) as a mechanism for metastatic colonization in breast cancer. Cancer Metastasis Rev.

[13] Aaltonen KE, Rosendahl AH, Olsson H, Malmström P, Hartman L, Fernö M (2014). Association between insulin-like growth factor-1 receptor (IGF1R) negativity and poor prognosis in a cohort of women with primary breast cancer. BMC Cancer.

[14] Kang HS, Ahn SH, Mishra SK, Hong KM, Lee ES, Shin KH, Ro J, Lee KS, Kim MK (2014). Association of polymorphisms and haplotypes in the insulin-like growth factor 1 receptor (IGF1R) gene with the risk of breast cancer in Korean women. PLoS One.

[15] Gee JM, Robertson JF, Gutteridge E, Ellis IO, Pinder SE, Rubini M, Nicholson RI (2005). Epidermal growth factor receptor/HER2/insulin-like growth factor receptor signalling and oestrogen receptor activity in clinical breast cancer. Endocr Relat Cancer.

[16] Mao Y, Shang Y, Pham VC, Ernst JA, Lill JR, Scales SJ, Zha J (2011). Polyubiquitination of insulin-like growth factor I receptor (IGF-IR) activation loop promotes antibody-induced receptor internalization and down-regulation. J Biol Chem.

[17] Sehat B, Andersson S, Vasilcanu R, Girnita L, Larsson O (2007). Role of ubiquitination in IGF-1 receptor signaling and degradation. PLoS One.

[18] Byron SA, Yee D (2003). Potential therapeutic strategies to interrupt insulin-like growth factor signaling in breast cancer. Semin Oncol.

[19] Yagishita N, Yamasaki S, Nishioka K, Nakajima T (2008). Synoviolin, protein folding and the maintenance of joint homeostasis. Nat Clin Pract Rheumatol.

[20] Yagishita N, Ohneda K, Amano T, Yamasaki S, Sugiura A, Tsuchimochi K, Shin H, Kawahara K, ohneda O, Ohta T, Tanaka S, Yamamoto M, Maruyama I, Nishioka K, Fukamizu A, Nakajima T (2005). Essential role of synoviolin in embryogenesis. J Biol Chem.

[21] Yamasaki S, Yagishita N, Nishioka K, Nakajima T (2007). The roles of synoviolin in crosstalk between endoplasmic reticulum stress-induced apoptosis and p53 pathway. Cell Cycle.

[22] Xu Q, Jiang Y, Yin Y, Li Q, He J, Jing Y, Qi YT, Xu Q, Li W, Lu B, Peiper SS, Jiang BH, Liu LZ (2013). A regulatory circuit of miR-148a/152 and DNMT1 in modulating cell transformation and tumor angiogenesis through IGF-IR and IRS1. J Mol Cell Biol.

[23] Ahmed A (2010). Prognostic and therapeutic role of nuclear factor-kappa B (NF-kappaB) in breast cancer. J Ayub Med Coll Abbottabad.

[24] Liu R, Liu C, Chen D, Yang WH, Liu X, Liu CG, Dugas CM, Tang F, Zheng P, Liu Y, Wang L (2015). FOXP3 controls an miR-146/NFκB negative feedback loop that inhibits apoptosis in breast cancer cells. Cancer Res.

[25] Koda M, Sulkowski S, Garofalo C, Kanczuga-Koda L, Sulkowska M, Surmacz E (2003). Expression of the insulin-like growth factor-I receptor in primary breast cancer and lymph node metastases: correlations with estrogen receptors alpha and beta. Horm Metab Res.

[26] Sheshadri N, Catanzaro JM, Bott AJ, Sun Y, Ullman E, Chen EI, Pan JA, Wu S, Crawford HC, Zhang J, Zong WX (2014). SCCA1/SERPINB3 promotes oncogenesis and epithelial-mesenchymal transition via the unfolded protein response and IL6 signaling. Cancer Res.

[27] Yang L, Han S, Sun Y (2014). An IL6-STAT3 loop mediates resistance to PI3K inhibitors by inducing epithelial-mesenchymal transition and cancer stem cell expansion in human breast cancer cells. Biochem Biophys Res Commun.

[28] Ombrato L, Malanchi I (2014). The EMT universe: space between cancer cell dissemination and metastasis initiation. Crit Rev Oncog.

[29] Hecht JL, Kotsopoulos J, Gates MA, Hankinson SE, Tworoger SS (2008). Validation of tissue microarray technology in ovarian cancer: results from the Nurses' Health Study. Cancer Epidemiol Biomarkers Prev.

[30] Wang S, Wu X, Zhang J, Chen Y, Xu J, Xia X, He S, Qiang F, Li A, Shu Y, Roe OD, Li G, Zhou JW (2013). CHIP functions as a novel suppressor of tumour angiogenesis with prognostic significance in human gastric cancer. Gut.

[31] Weichert W, Röske A, Gekeler V, Beckers T, Ebert MP, Pross M, Dietel M, Denkert C, Röchen C (2008). Association of patterns of class I histone deacetylase expression with patient prognosis ingastric cancer: a retrospective analysis. Lancet Oncol.

[32] Jia L, Xing J, Ding Y, Shen Y, Shi X, Ren W, Wan M, Guo J, Zheng S, Liu Y, Liang X, Su D (2013). Hyperuricemia causes pancreatic β-cell death and dysfunction through NF-κB signaling pathway. PLoS One.

[33] Wang S, Gong Z, Chen R, Liu Y, Li A, Li G, Zhou J (2009). JWA regulates XRCC1 and functions as a novel base excision repair protein in oxidative-stress-induced DNA single-strand breaks. Nucleic Acids Res.

