# Supplementary material for: HRD1 suppresses the growth and metastasis of breast cancer cells by promoting IGF-1R degradation
Source: Oncotarget. 2015 Oct 19;6(40):42854–67. doi: 10.18632/oncotarget.5733 (PMC4767476; doi:10.18632/oncotarget.5733)
Supplement: Supplementary file 1 [file oncotarget-06-42854-s001.pdf]

SUPPLEMENTARY FIGURES

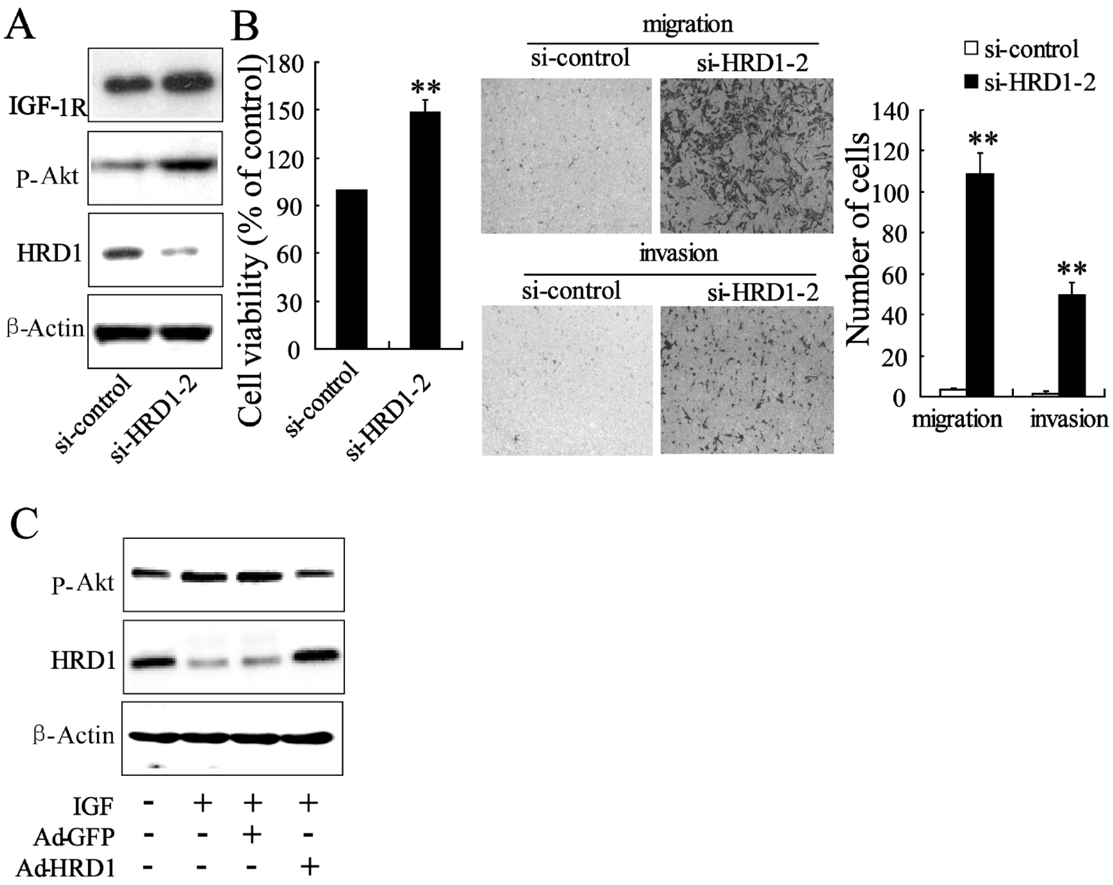

Supplementary Figure S1: Knockdown of HRD1 increased IGF1R expression and promoted MCF7 cell growth, migration, and invasion. A. The protein levels of HRD1, IGF-1R and *P*-Akt were measured by immunoblotting in HRD1 siRNA (si-HRD1-2) and their respective control transfected MCF-7 cells. **B.** Cell viability, migration, and invasion were measured si-HRD1-2 and their respective control transfected MCF-7 cells. **C.** MCF7 cells were infected with lentivirus of HRD1 for 48 h, and then treated with IGF (30 ng/ml), followed by immunoblotting. $^{**}P < 0.01$, compared to control.

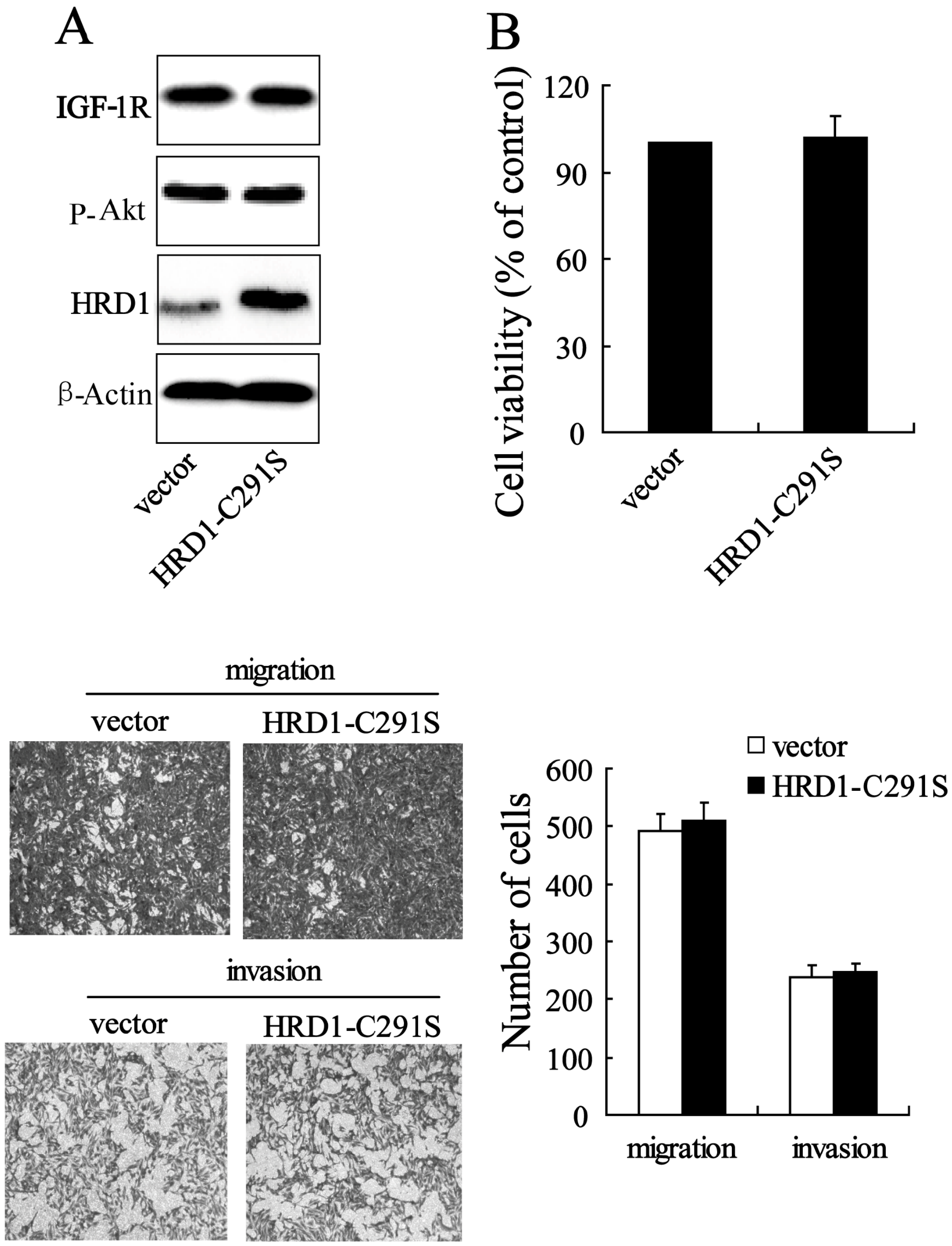

Supplementary Figure S2: Overexpression of a dominant-negative HRD1 mutant (C291S) had no effect on IGF1R expression and MB231 cell growth, migration, and invasion. A. The protein levels of HRD1, IGF-1R and *P*-Akt were measured by immunoblotting in overexpression of a dominant-negative HRD1 mutant (C291S) and their respective control transfected MB231 cell. **B.** Cell viability, migration, and invasion were measured in overexpression of HRD1-C291S and their respective control transfected MB231 cell.

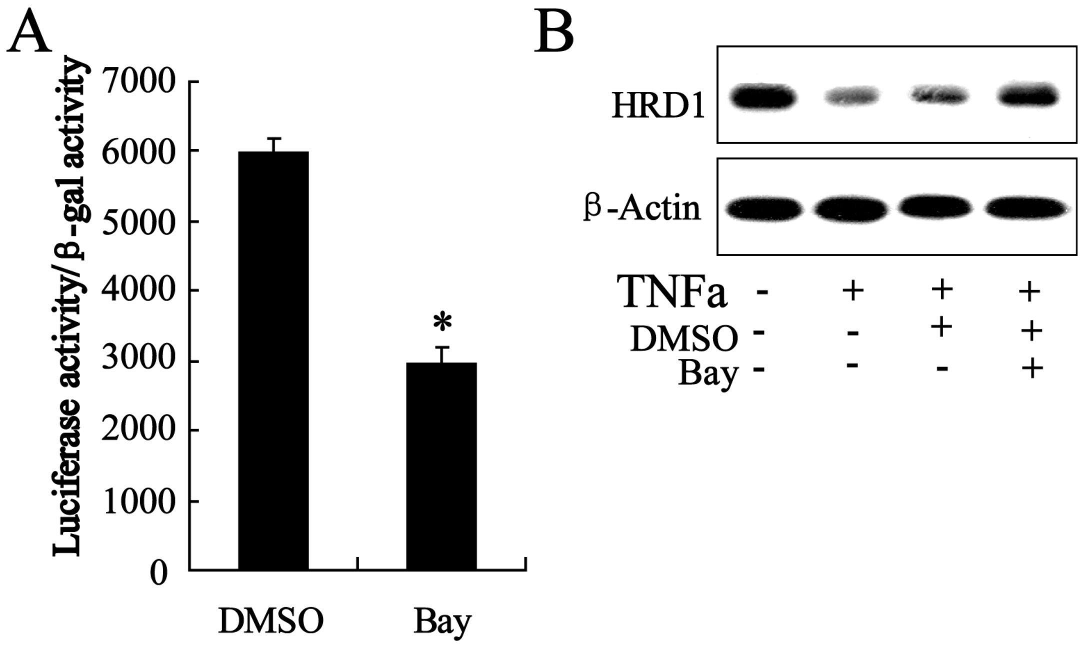

Supplementary Figure S3: TNFa inhibited HRD1 expression by activation of NF-κB. A. MCF-7 cells were treated with Bay 11-7082 (5 μmol/L) for 24 h, then, the NF-κB transcriptional activity was measured. **B.** MCF-7 cells were pretreated with Bay 11-7082 (5 μmol/L) for 2 h, and treated with IL-6 (50 ng/ml) for another 24 h. Then, HRD1 protein level was measured. *$P < 0.05$, compared to control.

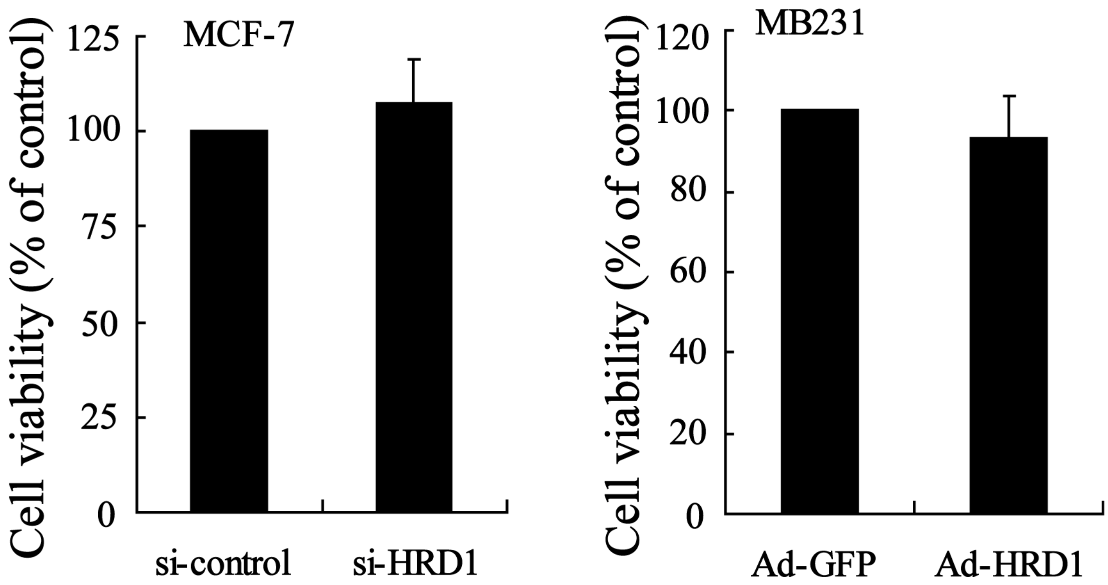

Supplementary Figure S4: The effect of HRD1 on cell viability was assessed. MCF-7 cells were transfected with si-control or si-HRD1 for 48 h, then, 1×10^5 cells in serum-free media were seeded in 24-well. After incubation for another 24 h, MTT assay was performed. MB231 cells were infected with Ad-GFP or Ad-HRD1 for 48 h, then, 1×10^5 cells in serum-free media were seeded in 24-well. After incubation for another 24 h, MTT assay was performed.
